# Human iPSC disease modelling reveals functional and structural defects in retinal pigment epithelial cells harbouring the m.3243A > G mitochondrial DNA mutation

**DOI:** 10.1038/s41598-017-12396-2

**Published:** 2017-09-26

**Authors:** Valeria Chichagova, Dean Hallam, Joseph Collin, Adriana Buskin, Gabriele Saretzki, Lyle Armstrong, Patrick Yu-Wai-Man, Majlinda Lako, David H. Steel

**Affiliations:** 1 Institute of Genetic Medicine, Newcastle University, Newcastle upon Tyne, NE1 3BZ United Kingdom; 2 Institute for Cell and Molecular Biosciences and The Ageing Biology Centre, Campus for Ageing and Vitality, Newcastle University, NE4 5PL, United Kingdom; 3 Cambridge Centre for Brain Repair, Department of Clinical Neurosciences, University of Cambridge, Cambridge, CB2 0PY United Kingdom; 4 MRC Mitochondrial Biology Unit, University of Cambridge, Cambridge, CB2 0XY United Kingdom; 5 Wellcome Trust Centre for Mitochondrial Research, Institute of Genetic Medicine, Newcastle University, Newcastle upon Tyne, NE1 3BZ United Kingdom; 6 NIHR Biomedical Research Centre at Moorfields Eye Hospital and UCL Institute of Ophthalmology, London, EC1V 2PD United Kingdom

## Abstract

The m.3243A > G mitochondrial DNA mutation was originally described in patients with mitochondrial encephalomyopathy, lactic acidosis, and stroke-like episodes. The phenotypic spectrum of the m.3243A > G mutation has since expanded to include a spectrum of neuromuscular and ocular manifestations, including reduced vision with retinal degeneration, the underlying mechanism of which remains unclear. We used dermal fibroblasts, from patients with retinal pathology secondary to the m.3243A > G mutation to generate heteroplasmic induced pluripotent stem cell (hiPSC) clones. RPE cells differentiated from these hiPSCs contained morphologically abnormal mitochondria and melanosomes, and exhibited marked functional defects in phagocytosis of photoreceptor outer segments. These findings have striking similarities to the pathological abnormalities reported in RPE cells studied from post-mortem tissues of affected m.3243A > G mutation carriers. Overall, our results indicate that RPE cells carrying the m.3243A > G mutation have a reduced ability to perform the critical physiological function of phagocytosis. Aberrant melanosomal morphology may potentially have consequences on the ability of the cells to perform another important protective function, namely absorption of stray light. Our *in vitro* cell model could prove a powerful tool to further dissect the complex pathophysiological mechanisms that underlie the tissue specificity of the m.3243A > G mutation, and importantly, allow the future testing of novel therapeutic agents.

## Introduction

The minimum prevalence of the m.3243A > G mutation in the mitochondrial gene *MT-TL1* encoding tRNA Leucine^(UUR)^ has been estimated at 3.5 per 100,000 in the UK population^1^. It can result in a broad phenotypic spectrum ranging from the classical syndrome of mitochondrial encephalomyopathy, lactic acidosis, and stroke-like episodes (MELAS) to varying combinations of neurological and ophthalmological manifestations^2,3^. The molecular mechanisms underlying the pathogenesis of the m.3243A > G mutation are complex and not fully understood, although a primary defect in mitochondrial translation is a possible explanation^4^. Typically, high energy demand organs are affected resulting in a multisystem presentation^5^, with 58% of patients having four or more clinical symptoms compared with 12% of patients who are monosymptomatic^6^. Ocular abnormalities are a common finding in patients with the m.3243A > G mutation with over half of all patients developing at least one ophthalmological manifestation, in particular progressive external ophthalmoplegia and ptosis, but also visual failure secondary to retinal dystrophy with pigmentary retinopathy, or more rarely optic atrophy^6–9^. Macular pigmentary abnormalities similar to those found in age-related macular degeneration (AMD) have been identified in about 1 in 5 of all m.3243A > G mutation carriers^8,10,11^, with atrophy of the retinal pigment epithelium (RPE) being the commonest finding^12,13^. Pale subretinal deposits have also been reported eccentrically around the areas of RPE atrophy, which are morphologically distinct from the typical central round drusen found in AMD^11,12,14^. In addition, the retinal deposits associated with the m.3243A > G mutation tend to be more hyper autofluorescent than those in AMD suggestive of a higher lipofuscin content.

The exact mechanisms leading to the observed changes in the retina in patients with the m.3243A > G mutation remain unknown. To circumvent for the lack of diseased human retinal tissues to study, we have generated human induced pluripotent stem cells (hiPSCs) from patients carrying the m.3243A > G mutation. These cells were then differentiated into RPE cells to dissect the downstream consequences on RPE function and the possible pathophysiological links that eventually result in progressive blindness.

## Results

### Derivation of patient hiPSCs with m.3243A > G mutation

**Figure 1:**
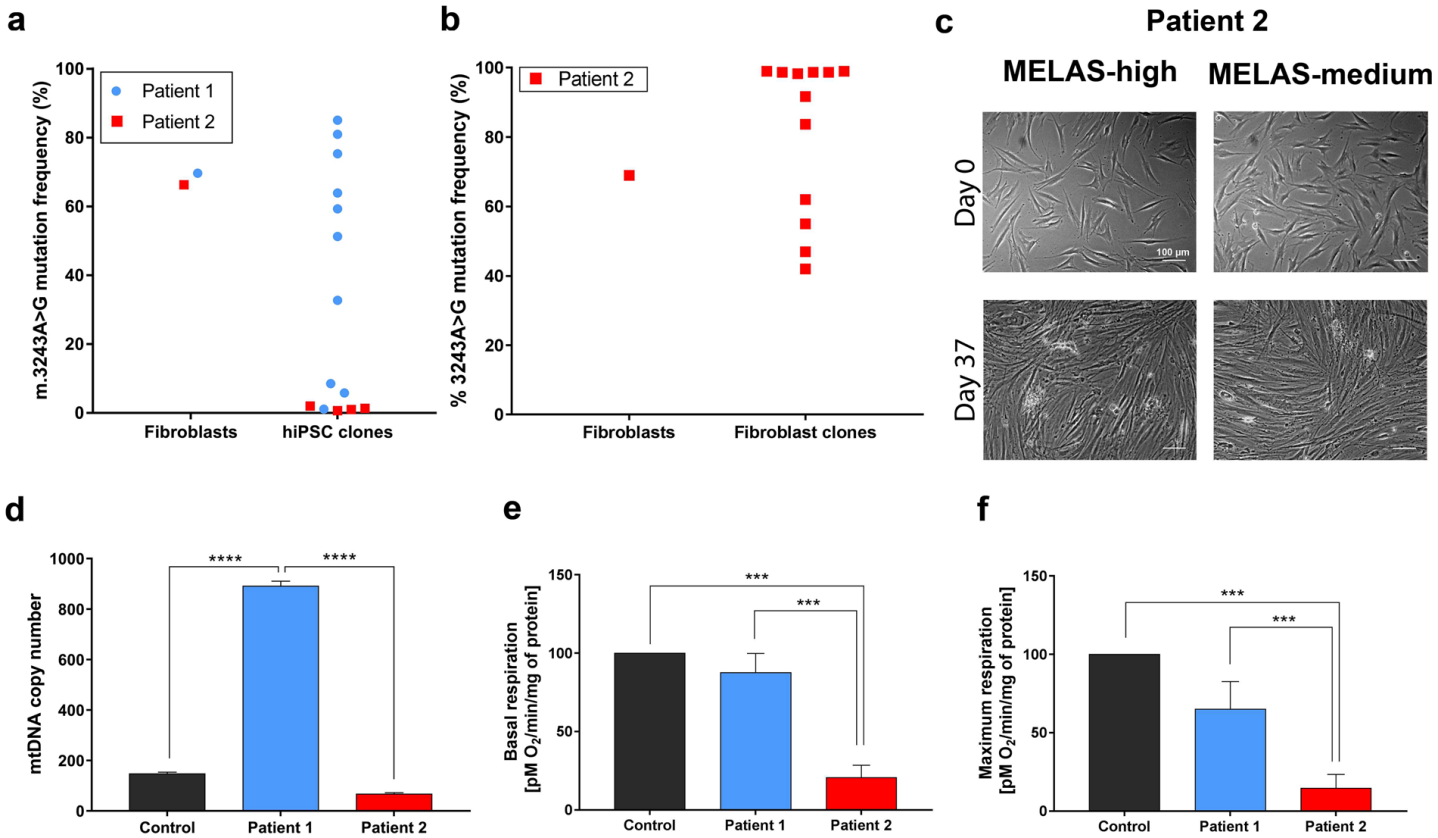
Derivation of hiPSCs with the m.3243A > G mutation. (**a**) Mutation frequency in MELAS-hiPSCs and parental fibroblasts. (**b**) Mutation load in Patient 2 fibroblasts and isolated fibroblast clones. (**c**) Representative images of the failed reprogramming attempt of Patient 2 fibroblasts. (**d**) mtDNA copy number in patients’ fibroblasts. Patient 1 cells had significantly increased levels of the mtDNA compared to the control and Patient 2 cells (n = 3). (**e**) Relative basal respiration in patient fibroblasts. Patient 2 cells had significantly decreased level of basal respiration cmparing to the control and Patient 1 cells (n = 3). (**f**) Relative maximal respiration in patient fibroblasts. Patient 2 cells had significantly decreased levels of maximal respiration compared to control and Patient 1 cells (n = 3). Data presented as mean ± SEM. Statistical significance was evaluated by one-way ANOVA followed by Tukey’s post hoc test. MELAS - mitochondrial encephalomyopathy, lactic acidosis, and stroke-like episodes; hiPSC – human induced pluripotent stem cell; mtDNA – mitochondrial DNA; SEM – standard error of the mean; ANOVA - analysis of variance.

Primary fibroblasts established from Patient 1 and Patient 2 were reprogrammed into hiPSCs using the Sendai virus-based system, which contains the following: polycistronic *hKlf4-hOct3/4-hSox2* (*KOS*), *hKlf4*, and *hc-Myc*. In addition, we attempted to derive hiPSC clones from fibroblasts derived from a third patient with m.3243A > G mutation, however, despite making multiple attempts using various Sendai-based reprogramming kits, these cells failed to give rise to hiPSCs (data not shown). The level of m.3243A > G heteroplasmy was established in parental fibroblasts and hiPSC clones from Patients 1 and 2. Fibroblast cells from both patients had similar levels of the mutation (69.67% ± 0.48 in Patient 1; 66.3% ± 0.57 in Patient 2; Fig. 1a). Once the hiPSC clones were isolated and sequenced, a wide range of heteroplasmy levels was found in Patient 1 hiPSCs ranging from 1.11% ± 1.09% to 85.05% ± 0.98%. Patient 2 hiPSC clones, however, had low mutation levels (ranging between 0.67% ± 1.15% and 2.00% ± 1.00%) (Fig. 1a). Sequencing these clones at later passages confirmed that they all contained wild-type mtDNA only. In order to isolate Patient 2 hiPSCs carrying the mutant mtDNA species, fibroblast cells were serially diluted and fibroblast clones with high-level (>90%) or mid-level (55–65%) of the m.3243A > G mutation were isolated. The sequencing results for the parental fibroblast population and the isolated clones are shown in Fig. 1b. Two high-level and two mid-level fibroblast clones were reprogrammed using the same method as in the original experiment. All these experiments failed to give rise to hiPSC colonies as illustrated in Fig. 1c. To provide a possible explanation for these results, fibroblast cells from Patient 2 were compared with the cells from Patient 1 across a number of parameters. Patient 1 fibroblast cells had significantly higher mtDNA copy number compared with Patient 2 and control cells (Fig. 1d), which could potentially indicate a more prominent compensatory response to the pathogenic m.3243A > G mtDNA mutation, in turn aiding with cellular reprogramming. In keeping with this hypothesis, fibroblast cells from Patient 1 had significantly higher levels of basal and maximal respiration compared with Patient 2 cells (Fig. 1e,f). Overall, these results show that Patient 1 and Patient 2 fibroblast cells differed in their bio-energetic profile, which potentially reduced the propensity of Patient 2 cells to give rise to hiPSCs with mutant mtDNA. Thus, subsequent experiments utilised hiPSCs derived from Patient 1 cells only.

### Characterisation of patient hiPSC clones

**Figure 2:**
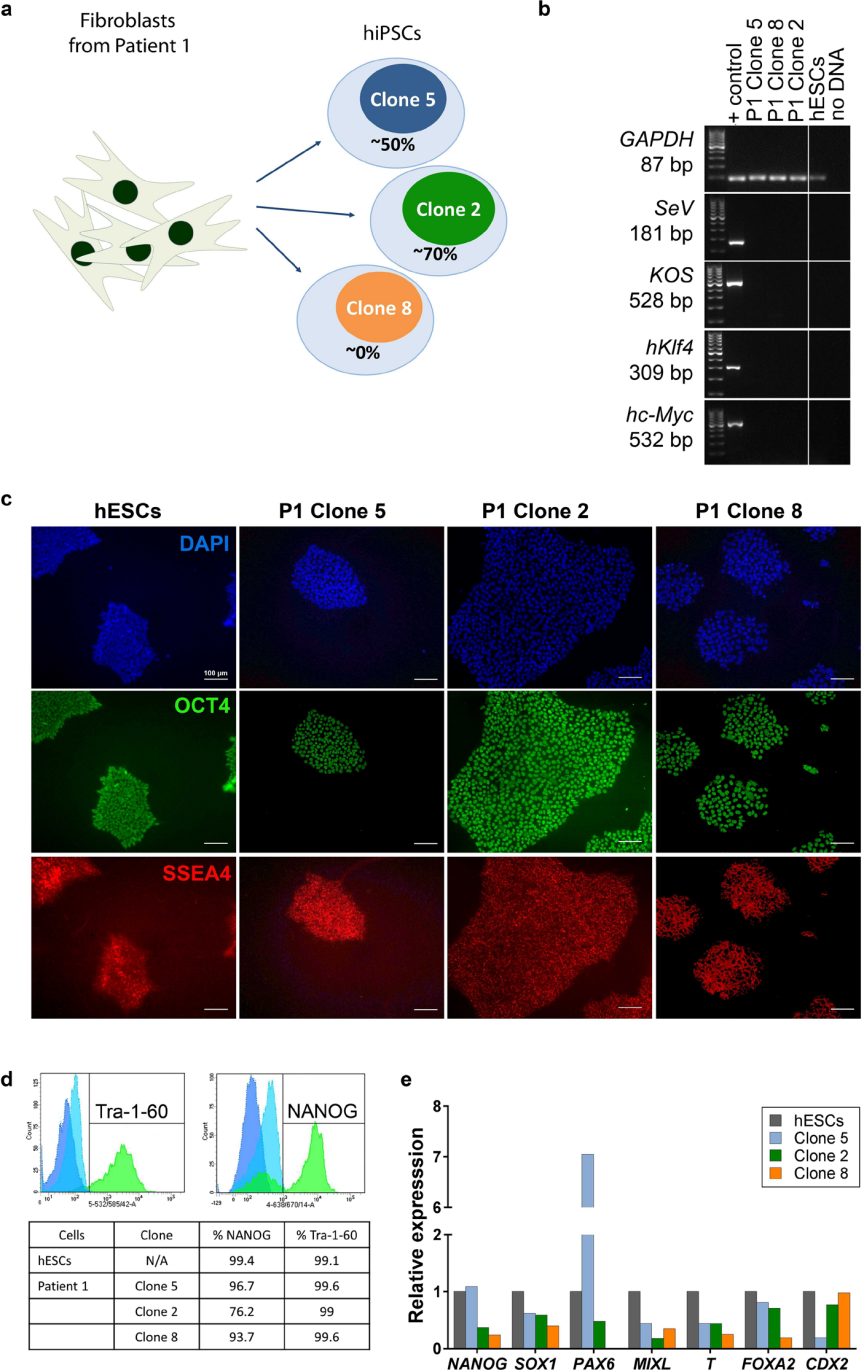
Validation of patient hiPSCs. (**a**) Patient 1 hiPSC clones selected for the analysis, indicating levels of heteroplasmic mtDNA. (**b**) hiPSC clones showed no sign of Sendai virus by passage 12, as verified by RT-PCR. Full length gel images are presented in Supplementary Figure S3. (**c**,**d**) hiPSCs expressed pluripotency-associated markers as validated by immunofluorescence and flow cytometric analysis (gating performed using isotype control and negative cell population, as shown in the figure). (**e**) EB culture of hiPSCs resulted in spontaneous differentiation into cells representative of the three embryonic germ layers (n = 1). Expression relative to hESCs. P – patient; hiPSC – human induced pluripotent stem cell; mtDNA – mitochondrial DNA; RT-PCR – reverse transcriptase polymerase chain reaction; EB – embryoid body; GAPDH – glyceraldehyde-3-phosphate dehydrogenase; SeV – Sendai viral vector genome; KOS – hKlf4-hOct3/4-hSox2; hKlf4 – human Kruppel-like factor 4; hc-MYC – human v-myc avian myelocytomatosis viral oncogene homolog; NANOG – Nanog homeobox; SOX1 – SRY (sex determining region Y)-box 1; PAX6 – paired box 6; MIXL – mix paired-like homeobox; T – T brachyury transcription factor; FOXA2 – forkhead box A2; CDX2 – caudal type homeobox 2; DAPI – 4′,6-diamidino-2-phenylindole; OCT4 – octamer binding factor 4; SSEA4 – stage-specific embryonic antigen 4.

Three hiPSC clones with varying levels of heteroplasmy were derived from Patient 1 fibroblasts and selected for further analyses. The levels of the m.3243A > G mutation were ~50% and ~70% in Clones 5 and 2, respectively, whereas Clone 8 was an isogenic control. These values correspond to average levels of mutated mtDNA at the time when the experiments were conducted (Fig. 2a). Prior to carrying out further experiments, it was first confirmed that the Sendai virus transgenes and the viral genome were cleared from the cytoplasm of the cells. RT-PCR showed that by passage 12, the virus was not present in the cells (Fig. 2b). Next, the hiPSCs were assessed for pluripotency-associated markers, specifically surface antigen SSEA4 and the nuclear localised transcription factor OCT4 by immunofluorescence (Fig. 2c), and TRA-1–60 and NANOG by flow cytometry, demonstrating that the majority of the cell population were positive for both markers (Fig. 2d). The developmental potential of the cells was accessed by differentiating them in monolayer cultures and allowing them to proliferate in bFGF-free medium for 2 weeks. Quantitative RT-PCR analysis demonstrated that the cells expressed markers associated with all three germ layers and extraembryonic tissues (Fig. 2e). Genomic SNP array was used to establish whether any significant genomic instabilities were present in parental fibroblasts and whether any *de novo* mutations occurred during the process of the reprogramming by testing both parental fibroblasts and hiPSC clones. Patient 1 fibroblasts showed no clinically significant imbalance. Clone 5 and Clone 8 had no major changes. Clone 2 had changes on chromosome 20 resulting in large scale deletion on 20p and duplication on 20q, which has previously been shown to be a common finding in hiPSCs^15^.

### Bio-energetic profile of patient hiPSC clones

**Figure 3:**
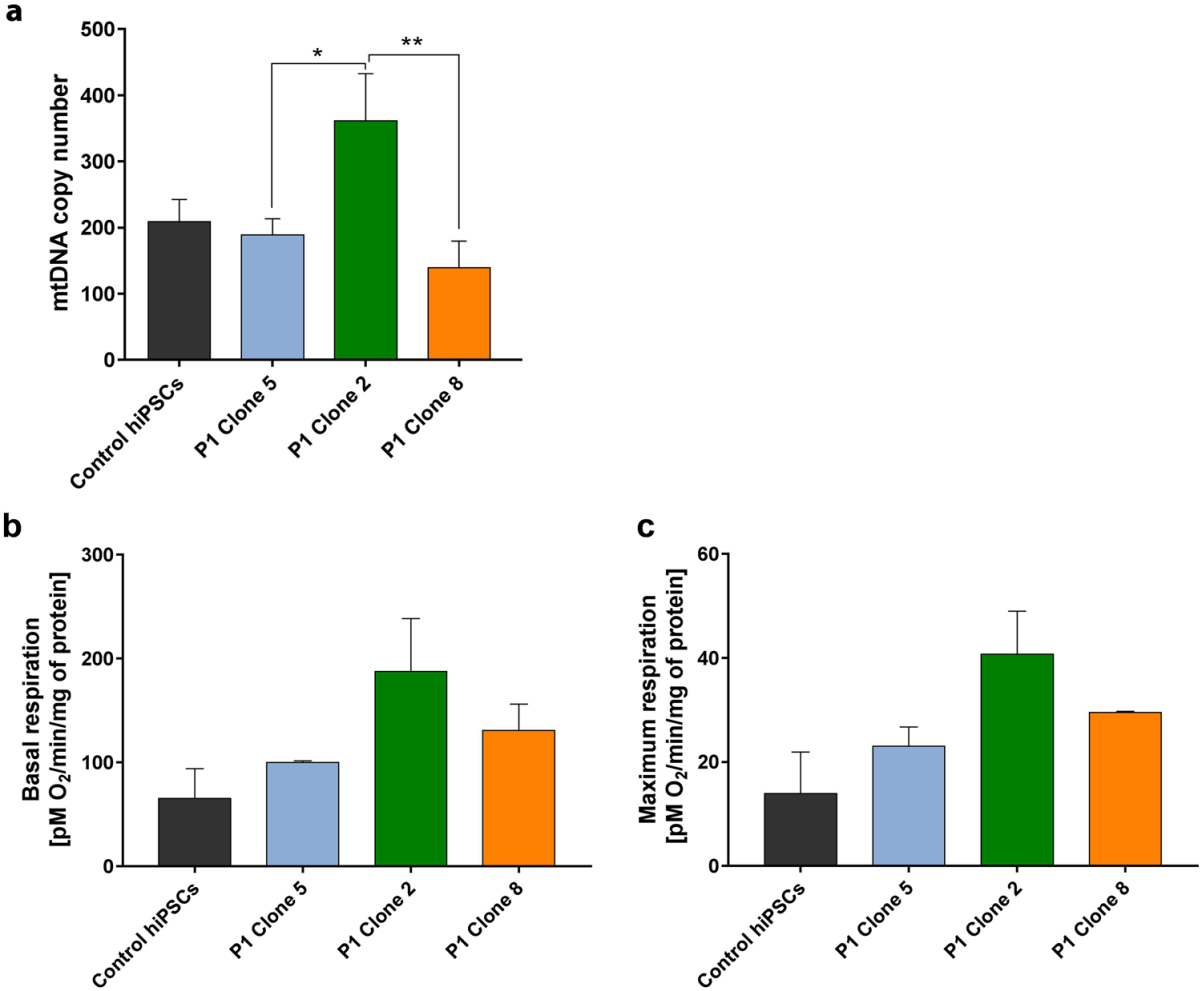
Characterisation of patient hiPSCs. (**a**) Clone 2-hiPSCs had significantly increased levels of mtDNA copy number, compared to Clone 5-hiPSCs and Clone 8-hiPSCs (n = 3). (**b**,**c**) There was no significant difference in basal or maximal respiration capacity between hPSCs (n = 3). Data presented as mean ± SEM. Statistical significance was evaluated by one-way ANOVA followed by Tukey’s post hoc test. P – patient; SEM – standard error of the mean; ANOVA - analysis of variance hiPSC – human induced pluripotent stem cell; mtDNA – mitochondrial DNA; hESC – human embryonic stem cell; hPSC – human pluripotent stem cell; SEM – standard error of the mean; ANOVA - analysis of variance.

It has been previously shown that hiPSCs with a high mutation load have increased levels of mtDNA copy number^16^. In this study the patient hiPSC clone with the highest level of mutated mtDNA (Clone 2) had significantly increased levels of mtDNA copy number compared with the other two clones (Fig. 3a). Despite the differences in mtDNA copy number, no significant variation in basal and maximal respiration was detected between hiPSC clones (Fig. 3b,c).

### Patient-derived hiPSCs give rise to RPE cells

**Figure 4:**
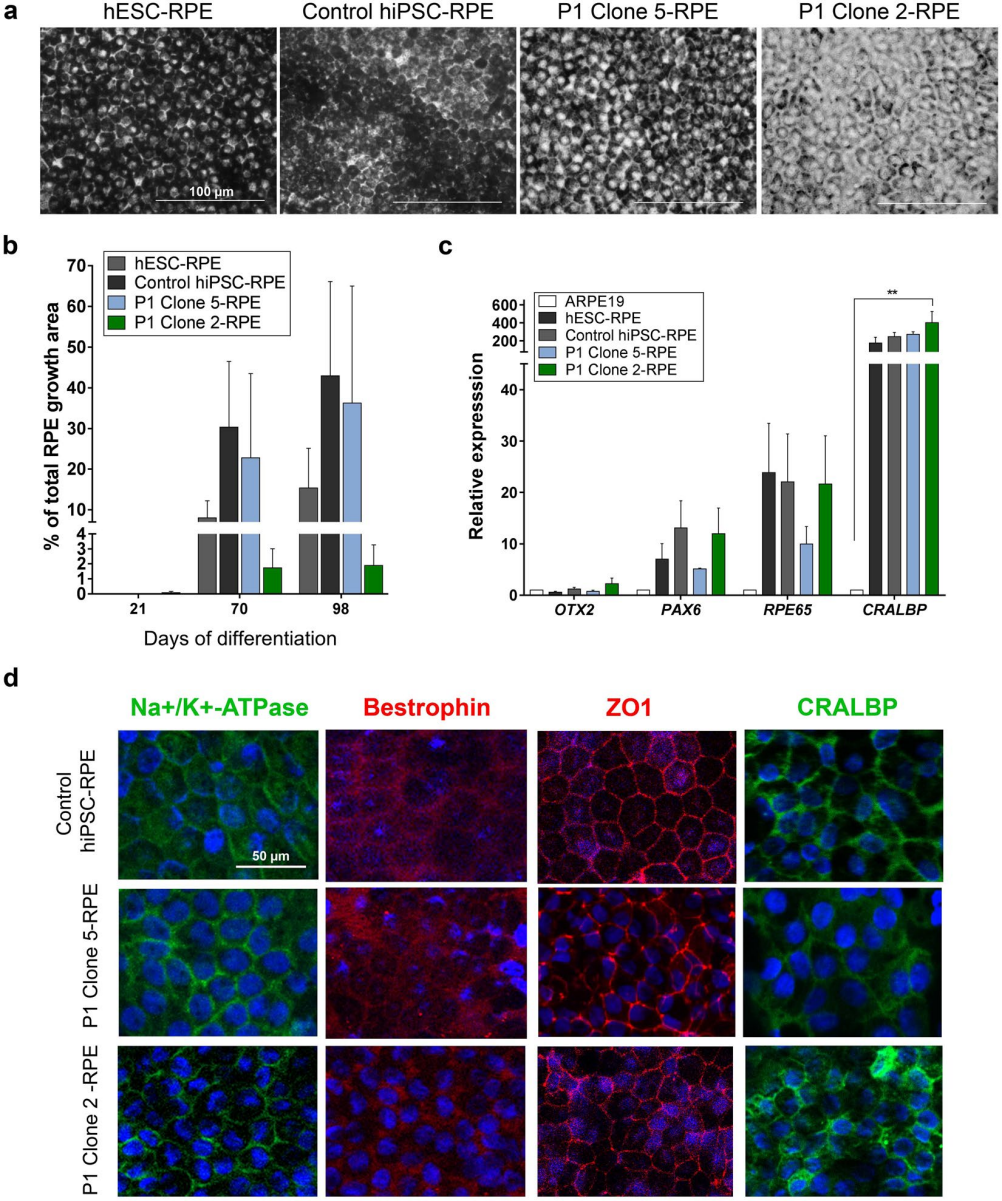
Characterisation of patient hiPSC-derived RPE cells. (**a**) Patient-derived RPE cells showed typical cobblestone morphology and appearance of pigment. (**b**) hPSCs showed similar RPE differentiation propensity as assessed by the percentage of growth area occupied by pigmented cells (n = 3). (**c**,**d**) RPE cells expressed early and mature RPE-related markers (n = 3). Expression relative to ARPE19 cells. Data presented as mean ± SEM. Statistical significance was evaluated by one-way ANOVA followed by Tukey’s post hoc test. P – patient; RPE – retinal pigment epithelium; OTX2 – orthodenticle homeobox 2; PAX6 – paired box 6; RPE65 – retinal pigment epithelium-specific protein 65 kDa; CRALBP – cellular retinaldehyde-binding protein; DAPI – 4′,6-diamidino-2-phenylindole; ZO1 – zonula occludens-1; SEM – standard error of the mean; ANOVA - analysis of variance.

hESCs, Control hiPSCs, Clone 5 and Clone 2 hiPSCs all gave rise to pigmented cells with typical cobblestone RPE morphology (representative images are shown in Fig. 4a). Interestingly, Clone 8 hiPSCs failed to differentiate into RPE cells. They formed cystic bodies, but no neural or retinal-like cells were produced after multiple differentiation attempts. Therefore, this clone was not used in subsequent differentiation experiments. No significant differences were seen between human pluripotent stem cell (hPSC)-derived RPE cells in the amount of time it took for the first pigment patches to appear (30.3 ± 5.6 days, mean ± standard deviation). There was an immediate observation that Clone 2-RPE produced the least pigmented cells although there was no significant difference in the ability of these clones to give rise to RPE patches over time (Fig. 4b). These differences did not affect the identity of the resulting cell population. All cells expressed genes associated with differentiating and mature RPE cells assessed by quantitative RT-PCR (Fig. 4c). Immunofluorescence was performed for detecting mature RPE markers (Na + /K + -ATPase, Bestrophin, and CRALBP localised to the membrane) and tight junction marker ZO1 showing labelling in the cell membrane tight junctions (Fig. 4d).

### Patient hiPSC-derived RPE cells show variable levels of heteroplasmy and mtDNA copy number

**Figure 5:**
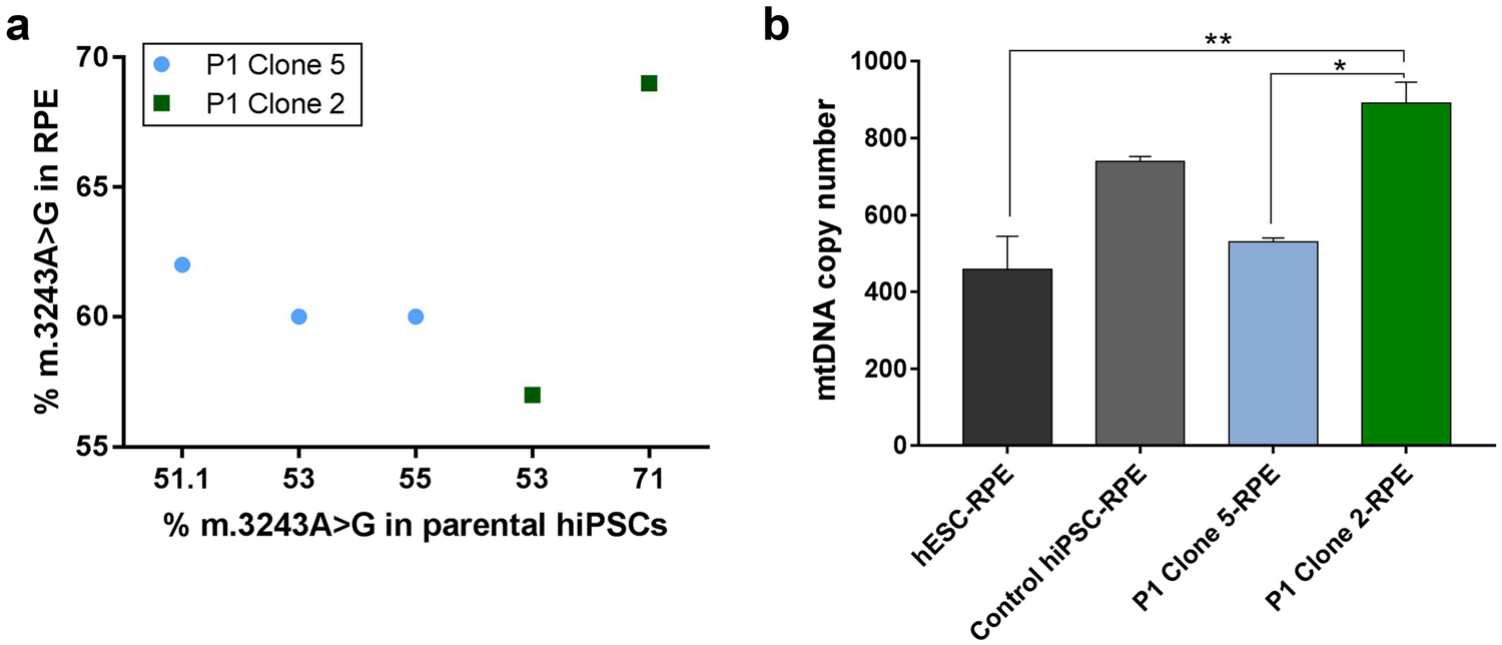
RPE cells show varying heteroplasmy and mtDNA copy number levels. (**a**) RPE cells derived from Clone 5 MELAS-hiPSCs showed increased levels of mutated mtDNA compared to parental hiPSCs. RPE cells from Clone 2 MELAS-hiPSCs had similar or reduced amount of mutated mtDNA. Clone 5-RPE were derived from hiPSCs at passage 17, 19, and 21. Clone 2-RPE were derived from hiPSCs at passage 14 and 19. On the X-axis the approximate levels of the mutation of the parental hiPSCs the RPE cells were derived from are indicated. (**b**) Clone 2-RPE had significantly increased levels of mtDNA copy number, compared to hESC-RPE and Clone 5-RPE cells (n = 2). Data presented as mean ± SEM. Statistical significance was evaluated by one-way ANOVA followed by Tukey’s post hoc test. P – patient; RPE – retinal pigment epithelium; hiPSC – human induced pluripotent stem cell; mtDNA – mitochondrial DNA; hESC – human embryonic stem cell; SEM – standard error of the mean; ANOVA - analysis of variance.

In order to investigate the cellular dynamics of the mitochondrial mutation change during the differentiation process, all clones were analysed for heteroplasmy levels and mtDNA copy number (Fig. 5a). RPE cells derived from Clone 5 had slightly increased levels of heteroplasmy compared with the original hiPSCs. Clone 2-RPE cells showed variable results from two separate differentiation experiments. There was no difference in heteroplasmy level of RPE cells compared with the original hiPSCs in the first differentiation experiment resulting in the derivation of RPE cells with ~70% of the mutation. However, the second differentiation resulted in the derivation of cells with ~57% m.3243A > G. The trend in the levels of the mtDNA in patient hiPSC-RPE cells mimicked the results obtained for hiPSC clones they were derived from. Clone 2-RPE had significantly higher number of mtDNA copies compared to RPE cells derived from Clone 5 and hESCs as shown in Fig. 5b. We also performed an ATP assay to evaluate the levels of oxidative phosphorylation (OXPHOS) and glycolysis in RPE cells. We found that there was a trend towards Clone 2-RPE cells having lowered levels of OXPHOS comparing to control RPE cells and Clone 5-RPE cells (Supplementary Figure S2).

### Patient-derived RPE cells show ultrastructural changes in melanosomes and mitochondria

**Figure 6:**
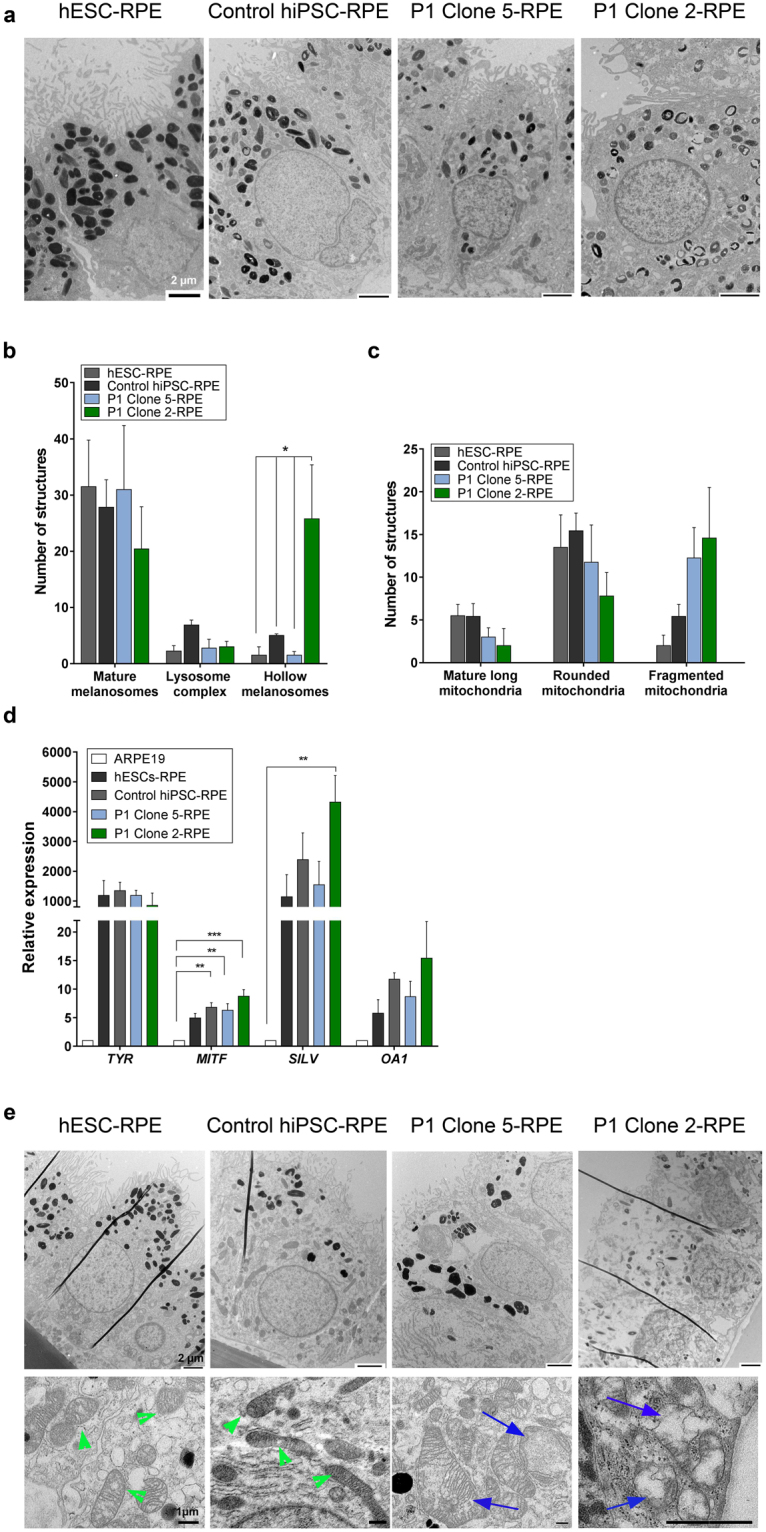
Ultrastructural examination of patient RPE cells. (**a**) Representative TEM images of RPE cells. Patient RPE cells with high level m.3243A > G have atypical melanosomes with hollow morphology. (**b**) Clone 2-RPE cells had significantly higher levels of hollow melanosomes. No difference was found in the numbers of mature melanosomes and melanosomes associated with lysosomes. Quantifications are based on cells from 3–7 random fields. (**c**) Quantifications of various types of mitochondria. No difference was found between cells. Quantifications are based on cells from 3–7 random fields. (**d**) Relative expression of genes associated with pigment synthesis. No difference was found between hPSC-derived RPE cells (n = 3). Expression relative to ARPE19 or human retina (*OA1*). (**e**) Aged patient RPE cells (3 months older). Clone 5-RPE had very large dysmorphic mitochondria with abnormal cristae. Clone 2-RPE had apoptotic nuclei, disturbed membranes, disorganised cytoplasm and indistinguishable mitochondria. Healthy mitochondria are indicated by green arrowheads and dysmorphic mitochondria are indicated by blue arrows in the bottom panel. Note: black vertical lines seen in hESCs-RPE, Control hiPSC-RPE and Clone 2-RPE are a processing artefact. Data presented as mean ± SEM. Statistical significance was evaluated by one-way ANOVA followed by Tukey’s post hoc test. TEM – transmission electron microscopy; RPE – retinal pigment epithelium; hESC – human embryonic stem cell; hiPSC – human induced pluripotent stem cell; TYR – tyrosinase; MITF – microphthalmia-associated transcription factor; SILV – silver locus protein homolog; SEM – standard error of the mean; ANOVA - analysis of variance.

RPE cells were imaged by transmission electron microscopy (TEM) in order to investigate their morphology and ultrastructure. There were striking differences in the morphology of RPE cells derived from patient Clone 2 hiPSCs, which had ~70% of the mutation (Fig. 6a). The Clone 2-RPE cells did not display the typical baso-apical distribution of organelles seen in normal RPE cells, where melanosomes are located apically. Furthermore, the mutant RPE cells exhibited underdeveloped microvilli and they contained melanosomes that were hollow inside. Clone 2-RPE cells had similar numbers of mature melanosomes and melanosomes associated with lysosomes, compared with hESC-RPE, Control hiPSC-RPE, and Clone 5-RPE. However, Clone 2-RPE contained significantly more hollow melanosomes with atypical morphology (Fig. 6b). This finding is in line with clinical observations that have shown that MELAS patients have areas of RPE depigmentation funduscopically. Mitochondrial morphology was also assessed. Although there was no statistical significance in the findings, there was a trend towards patient-derived RPE cells having less mature and more fragmented/damaged mitochondria, which could be a direct result of m.3243A > G mutation (Fig. 6c). Quantitative RT-PCR results of genes related to pigment synthesis did not reveal differences between patient and control RPE cells indicating that the morphological changes were unlikely to be due to defect in pigment formation (Fig. 6d). Three months after the first TEM images were taken, another set of RPE cells that were grown in parallel to the first set of cells were imaged in order to confirm whether the cells retained their pathological morphology over time. As seen in Fig. 6e, hESC- and Control hiPSC-derived RPE cells maintained their baso-apical polarity and other features typical of RPE cells. Interestingly, Clone 5-RPE cells contained swollen fragmented mitochondria. Clone 2-RPE cells looked apoptotic across all images. Additionally, Clone 2 cells had disturbed membranes, dysmorphic mitochondria, underdeveloped microvilli, vacuoles and a mix of melanosomes with various morphologies. Due to the difficulty of distinguishing between organelles it was not possible to replicate the quantifications performed for the first set of TEM images. However, from these images it can be seen that patient cells looked degenerative, indicating potential effect of the mutation on their ability to support RPE survival.

### Patient-derived RPE cells show functional defects

Cells were assessed for their ability to perform functions that are known to be typical for RPE cells *in vivo*. Trans-epithelial resistance (TER) measurements were used as an indirect measurement of the ability of the cells to form the outer blood-retinal barrier by detecting functional tight junctions. No significant difference was found between the cells (Supplementary Figure S1).

**Figure 7:**
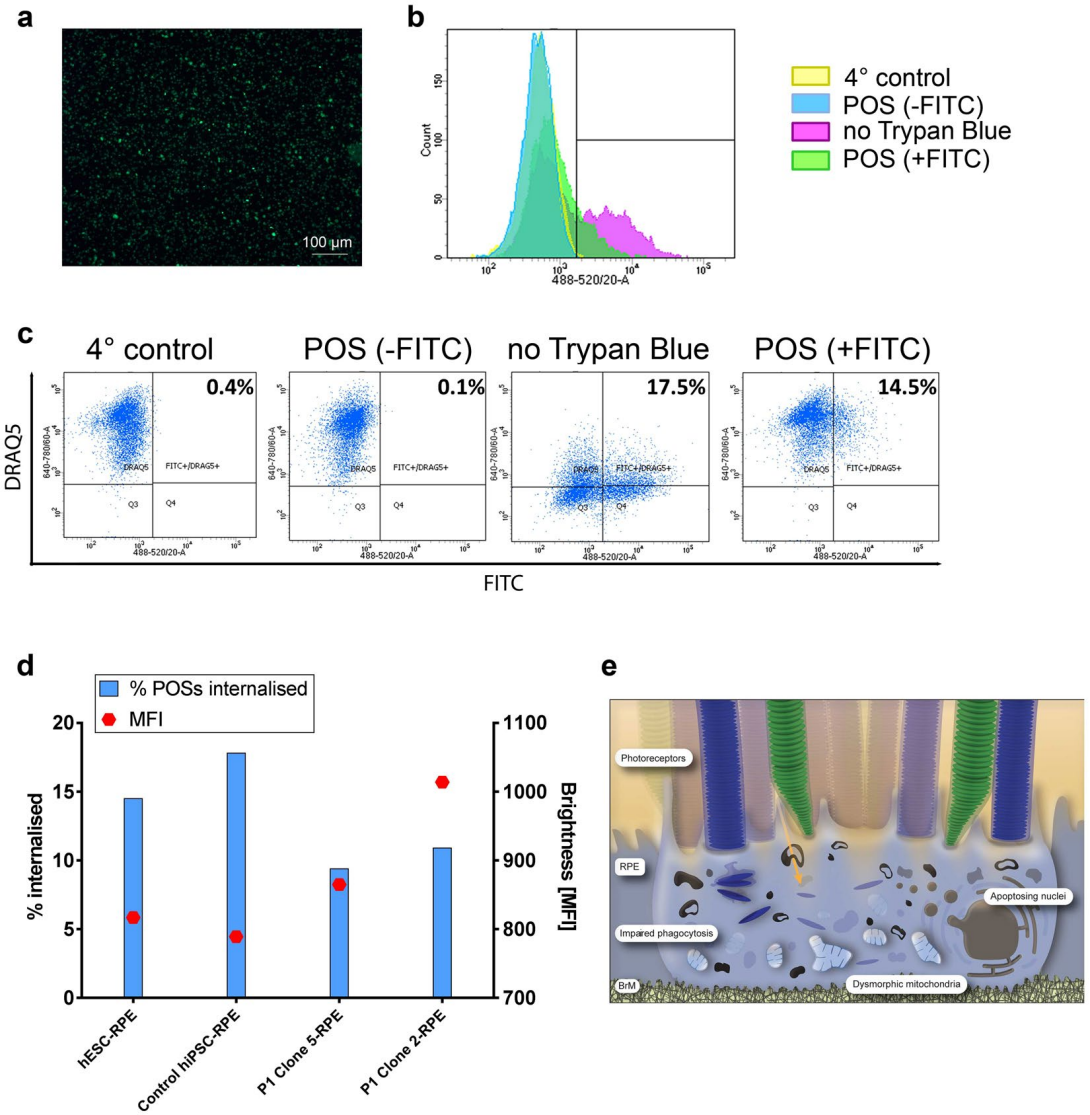
Functional characteristics of patient RPE cells. (**a**) FITC-labelled POS. (**b**) Histogram representation of the control experiments. (**c**) Blocking phagocytosis by incubating the cells at 4 °C, feeding cells with POS without FITC-labelling, performing the experiment without quenching extracellular FITC-labelled POS, example of the results with cells fed with FITC-labelled POS and quenching extracellular POS with Trypan blue, showing its effect. (**d**) Phagocytosis experiment results showed that hESCs and control hiPSCs had a higher number of cells that were able to phagocytose POS. Patient cells that were able to phagocytose had the ability of ingesting more POS per cell, as determined by MFI values. All cells were matched with regards to the length of differentiation time and were all ~6.5 months old. (**e**) Schematic representation of areas of RPE cells affected by the mutation, illustrating disturbed phagocytosis of POS, hollow melanosomes, apoptotic nuclei, and dysmorphic mitochondria. Orange arrow depicts light path. Data presented as mean ± SEM. Statistical significance was evaluated by one-way ANOVA followed by Tukey’s post hoc test. FITC – fluorescein isothiocyanate; POS – photoreceptor outer segments; hESC – human embryonic stem cell; hiPSC – human induced pluripotent stem cell; MFI – median fluorescence intensity; RPE – retinal pigment epithelium; BrM – Bruch’s membrane. SEM – standard error of the mean; ANOVA - analysis of variance.

The ability to phagocytose photoreceptor outer segments (POS) is one of the main functions of RPE cells. An assessment of the ability of the cells to phagocytose was carried out by feeding the RPE cells with FITC-labelled POS and using flow cytometry to quantify the results. FITC-labelling of POS was confirmed with immunofluorescence (Fig. 7a). A number of control experiments were set up to ensure the robustness of the data. Firstly, cells were kept at 4 °C in order to block their ability to phagocytose^17^. Trypan blue was used to quench any extracellular fluorescence produced by POS bound to the extracellular membrane but not ingested, giving false positive results on the cytometer^18^. Cells were also fed with unlabelled POS to assess background fluorescence. Representative images of the settings results are shown in Fig. 7b,c. Two main conclusions were drawn from the results of the experiment. The percentage of FITC-positive cells indicates the number of cells within the population that ingested POS. Median fluorescence intensity (MFI) represents the amount of POS ingested by individual cells as an indirect indication of cell-surface receptor density involved in phagocytosis^18^. The MFI values were obtained by subtracting background fluorescence values in the FITC channel in the absence of labelled POS. The results of the experiment showed that there was approximately 1/3 less cells within the population of patient-derived RPE cells that were able to phagocytose (hESCs-RPE 14.5%; Control hiPSCs-RPE 17.8%; Clone 5-RPE 9.4%; Clone 2-RPE 10.9%). On the other hand, there was a population of phagocytosing cells that were ingesting higher numbers of POS as indicated by higher MFI values. Quantifications are shown in Fig. 7d. Schematic illustration of the RPE pathology is shown in Fig. 7e.

## Discussion

Outer retinal involvement is an important cause of progressive irreversible visual loss among m.3243A > G mutation carriers, but the underlying disease mechanisms still remain unclear, precluding a targeted therapeutic approach^10–12,14,19^. In the current study, we found that RPE cells derived from hiPSCs harbouring the m.3243A > G mtDNA mutation showed abnormal ultrastructural and functional defects pointing towards a direct pathogenic effect on this particular retinal cell type. RPE cells derived from patient hiPSCs were comparable with controls in terms of the expression of early and mature RPE markers, barrier properties, and genes associated with pigmentation. However, RPE cells with ~50–70% heteroplasmy showed ultrastructural changes in their mitochondria and melanosomes, and additionally they showed defects in phagocytosis of photoreceptor outer segments.

Unlike Patient 1, fibroblasts from Patient 2 failed to give rise to mutant hiPSC clones with m.3243A > G. Previous studies that reprogrammed cells with mtDNA mutations reported generation of homoplasmic, heteroplasmic and mutation free hiPSC clones^16,20–22^. Despite the significant effort of attempting to isolate hiPSC clones carrying m.3243A > G mutation from Patient 2 fibroblasts, including reprogramming clonally isolated fibroblasts, we were not able to isolate hiPSCs with m.3243A > G from this patient. In our study we showed that the primary fibroblast cells from Patient 2 had significantly lower mtDNA copy number and levels of cellular respiration compared with Patient 1 cells, possibly because the latter were compensating better for the defects in translation by increasing the amount of mtDNA, thereby allowing for more successful cellular reprogramming^23^. The role of metabolic profile in cellular reprogramming is a subject of increasing interest. Mitochondria undergo a number of changes that are required for successful reprogramming. Somatic cell reprogramming is accompanied by lowering of mitochondrial content, changes in regulation of some mitochondrial genes, metabolic resetting by switching from OXPHOS to glycolysis, and remodelling of mitochondrial shape from immature round to more elongated with complex cristae^24,25^. One study showed that the bio-energetic profile of parent cells affects their ability to generate hiPSCs and cells with higher propensity for glycolytic metabolism reprogram with higher efficiency^26^. Although speculative, it is plausible that Patient 2 cells were unable to undergo a switch between OXPHOS and glycolysis to reach successful reprogramming^27^. Interestingly, additional fibroblasts from a third patient with m.3243A > G mutation failed to reprogram altogether, highlighting the difficulties in induction of pluripotency in these cells. This could have been due to high levels of the mutation in these cells, comparing to the other two patients (~87% in Patient 3 vs. ~70% in Patient 1 and ~66% in Patient 2), which has been previously described to result in a reprogramming block^22^. Other studies that investigated mutations in mtDNA have similarly used low number of cell lines^21,28–30^. The effect of mtDNA mutations on the process of the reprogramming is a growing area of research that needs to be investigated further, as it may have potential therapeutic significance in the generation of disease free cell lines.

The hiPSCs that were used in this study expressed pluripotency-associated markers and they were able to differentiate into all three embryonic germ layers. Clone 2 cells from Patient 1 had changes on chromosome 20. These are common during both early and late passaging and indeed 25% of otherwise karyotypically normal cells have gain of 20q due to this region containing a highly repetitive sequence predisposing it to structural rearrangements^15^. Interestingly, hESCs with amplification of 20q11.21 acquired during long-term culture did not have any noticeable differences compared with normal cells with regards to morphology, pluripotency marker expression and growth^31^. In addition, a gain of 20q does not always imply overexpression of the genes located in that chromosomal region^15,31^. We chose Clone 2 hiPSCs for the differentiation experiments because these cells had the highest level of heteroplasmy across the hiPSC clones derived from this patient that survived and gave rise to RPE, making it an interesting comparison to the clone with lower levels of m.3243A > G, and hESCs and control hiPSCs. Clone 2 hiPSCs had significantly higher mtDNA copy number. However, there was no difference between hiPSC clones in terms of cellular respiration and cells with m.3243A > G were able to differentiate successfully into RPE cells. We tested the cells for the expression of the key RPE markers and found that they retained their RPE identity over time. This is different from the findings by Zhao and colleagues, who showed that ablation of RPE mitochondrial oxidative phosphorylation leads to de-differentiation of the cells^32^. It is possible that since we did not observe complete loss of OXPHOS in patient-derived RPE cells, cells with the m.3243A > G mutation with the heteroplasmy levels described do not acquire the more profound alterations in RPE function, but do result in the abnormalities we observed. Investigating the effects of the mutation further by using genetic manipulation techniques, such as mitoTALENs (mitochondria-targeted transcription activator-like effector nucleases)^33^ would provide further insight into the pathological mechanism of the m.3243A > G.

The key observation in this study was the striking differences in the morphology of melanosomes in patient-derived RPE cells. The cells had significantly increased levels of hollow melanosomes suggestive of a process of degradation and lacked microvilli which are vital for RPE-photoreceptors crosstalk, apical transport and visual cycle^34^. Furthermore, when samples were analysed again three months later, the m.3243A > G hiPSC-RPE cells contained swollen mitochondria, loss of cristae morphology, vacuoles, disrupted membrane integrity and Clone 2-RPE had apoptotic nuclei, indicative of a progressive neurodegenerative phenotype. Melanin is mainly synthesised in postnatal RPE and there are studies that show that there is general decrease in the number of pigment granules per macular RPE with age^35^. This is accompanied by an increasing association of melanosomes with lysosomes and the formation of lipofuscin, as a result of the inability of RPE to cope with phagosomal load^35^. The aetiology of the hollow melanosomes observed in differentiated RPE cells harbouring the m.3243A > G mutation need to be investigated further. Two possibilities include the melanosomes being immature with incomplete formation or alternatively, they are mature melanosomes that have undergone degradation. The first hypothesis was ruled out after establishing that the cells had comparable levels of expression of genes involved in melanogenesis, including *TYR*, *MITF, SILV*, and *OA1*. This is of interest as it has previously been suggested that some melanin biosynthetic gene variants are risk factors for AMD^36^. The melanosomes observed in Clone 2-RPE had a striking resemblance to degrading melanosomes found in ageing rhesus monkeys (*Macaca mulatta*)^37^. RPE cells in rhesus monkeys contain hollow melanosomes which were described as a new class of organelles that degrade their own melanin via lysosomal and autophagic actions^37^. They suggested that such morphological changes were the result of vulnerability to stress and pointed out that melano-lysosomes, were associated with early age related maculopathy. This is consistent with mitochondrial stress secondary to m.3243A > G mutation. A recent study showed that treating RPE cells with AICAR (5-aminoimidazole-4-carboxamide ribonucleotide), an activator of AMP-dependent protein kinase (AMPK) activity, together with proteasome inhibition leads to decreased levels of pigmentation^38^. Depletion of ATP leads to AMPK activation^39^, therefore it could be hypothesised that decreased ATP levels due to the mtDNA mutation could lead to a reduction of pigmentation levels, an assumption that could be investigated as part of future work. The exact mechanism of maculopathy as a result of accumulation of hollow melanosomes is not known; however it is plausible that the retinal atrophy in patients with the m.3243A > G might have common pathways with the pathological reduction of pigment seen in patients with AMD^34^. It could be hypothesised that these changes lead to the inability of the melanosomes to perform one of their key functions namely absorption of stray light.

TEM images also showed that patient RPE cells had swollen fragmented mitochondria with disrupted cristae. Interestingly, RPE cells from aged donors and AMD patients have been shown to contain disrupted mitochondrial networks that are unable to cope with cellular energy demands increasing their susceptibility to oxidative stress^40^. Stress-induced mitochondrial hyperfusion (SIMH) is an important cellular compensatory mechanism triggered by various stress stimuli, and in RPE of aged rhesus monkeys, elevated mitochondrial fusion is associated with increased resistance to apoptosis and autophagy^41,42^. Mitochondrial cristae sequester pro-apoptotic cytochrome c molecules and disruption of the tight junctions, in addition to reduced SIMH, are both likely to potentiate RPE cell death in the presence of the biochemically deleterious m.3243A > G mutation.

A second key observation was the inability of RPE cells harbouring the m.3243A > G mutation to efficiently phagocytose photoreceptor outer segments. Photoreceptor outer segments are continually renewed in a balanced fashion being replaced by newly formed outer segment disks. The daily renewal of POS is approximately 10% of their total length and each RPE cell is responsible for the removal of POS from 30–40 photoreceptors by the process of heterophagy^43^. By eliminating POS, the RPE protects photoreceptors from oxidative damage and accumulation of lipofuscin, thereby maintaining a healthy sub-retinal environment^44^. The proportion of Clone 2-RPE cells showing active phagocytosis was reduced by approximately a third compared with hESC-RPE cells and Control hiPSC-RPE cells, but MFI values suggested that the cells that were able to ingest POS were potentially more efficient than controls. The MFI data therefore suggests that the Clone 2-RPE cells that were phagocytosing did not have defects in the ability to recognise and engulf POS. Indeed, the values were comparable to the findings by another group that showed similar results in their control RPE cells derived from hiPSCs with the same flow cytometry technique^18^. Based on our findings, we speculate that there were a smaller number of functional RPE cells within the total population of Clone 2-RPE cells possibly due to intracellular variation in heteroplasmy levels. Healthier cells were compensating for defects in phagocytosis in dysfunctional cells, potentially resulting in additional pressure to degrade POS in phago-lysosomes. This is especially important because lysosomal overload has been shown to be associated with lipofuscin accumulation, vacuolation and phagocytosis disruption in RPE cells^45,46^. Interestingly, the effect of lipofuscin accumulation on the ability of cells to perform phagocytosis is exacerbated by the presence of mtDNA mutations with lower ATP production^47^. Disturbances in the ability of RPE to phagocytose could have major impact on retinal health. Defects in RPE phagocytosis lead to development of retinitis pigmentosa, Usher syndrome and AMD by a variety of different mechanisms^48–50^. Interestingly, clinical observations showed that some patients with m.3243A > G accumulate hyper-autofluorescent material in the subretinal space^12^. Histopathological studies on post-mortem eyes of MELAS patients have also indicated an increased presence of lipofuscin in RPE suggestive of a defect in POS phagocytosis^19,51^. A study of mtDNA from RPE cells of AMD donors found that there were a number of hot spots within the mtDNA where the mutations were most common and strikingly one of them was in the same mtDNA region as the m.3243A > G mutation suggesting possible common pathological pathways^52^. Golestaneh and colleagues found that RPE cells differentiated from iPSCs from AMD patients contained disintegrated mitochondria, reduced mitochondrial activity, and dysfunctional SIRT1/PGC-1α pathway further supporting the involvement of mitochondria in AMD disease progression^53^. There were striking similarities between our patient hiPSC-derived RPE cells and the appearance of RPE cells in post-mortem retinal sections obtained from m.3243A > G mutation carriers, in which depigmented RPE cells and abnormal mitochondria have been observed^19,51^. These findings complement our findings on the disturbances in phagocytosis and the possible accumulation of undigested materials, including melanosomes and swollen mitochondrial fragments, inside the cells, leading to cell death. Both RPE and photoreceptors are vulnerable even at low levels of heteroplasmy due to their localisation in a highly oxidative environment, however there is no evidence to clearly suggest whether it is RPE or photoreceptors, or both that are affected first in MELAS^12,54^. The retinal pathology observed in patients carrying the m.3243A > G mutation have intriguing overlapping features with the geographic retinal atrophy that is characteristic of AMD, the commonest cause of blindness in the developed world^11,55,56^. Currently, AMD is considered to be primarily a disease of RPE that in turn leads to secondary photoreceptor degeneration^57^. There is mounting evidence pointing towards oxidative stress and mitochondrial dysfunction as central pathological factors in the aetiology of AMD^58,59^. Investigating the pathophysiology of the outer retinal changes and specifically RPE in patients with m.3243A > G mtDNA mutation thus provides further circumstantial evidence pointing towards the role of mitochondrial dysfunction in patients with AMD.

In summary, we provide corroborative evidence that the m.3243A > G mutation has a detrimental impact on both the structural organisation and functional properties of RPE cells, in particular phagocytosis. To the best of our knowledge, we are first to report generation of RPE cells from hiPSCs carrying m.3243A > G mutation. Further work is needed to investigate how a primary defect in mitochondrial translation relates to the pathological changes and ultimately, the development of clinically significant retinal degeneration. The identification of mitochondrial pathways involved could pave the way for targeted therapeutic modulation to halt or slow the process leading to blindness in this patient group.

## Methods

### Patients and healthy controls information

**Figure 8:**
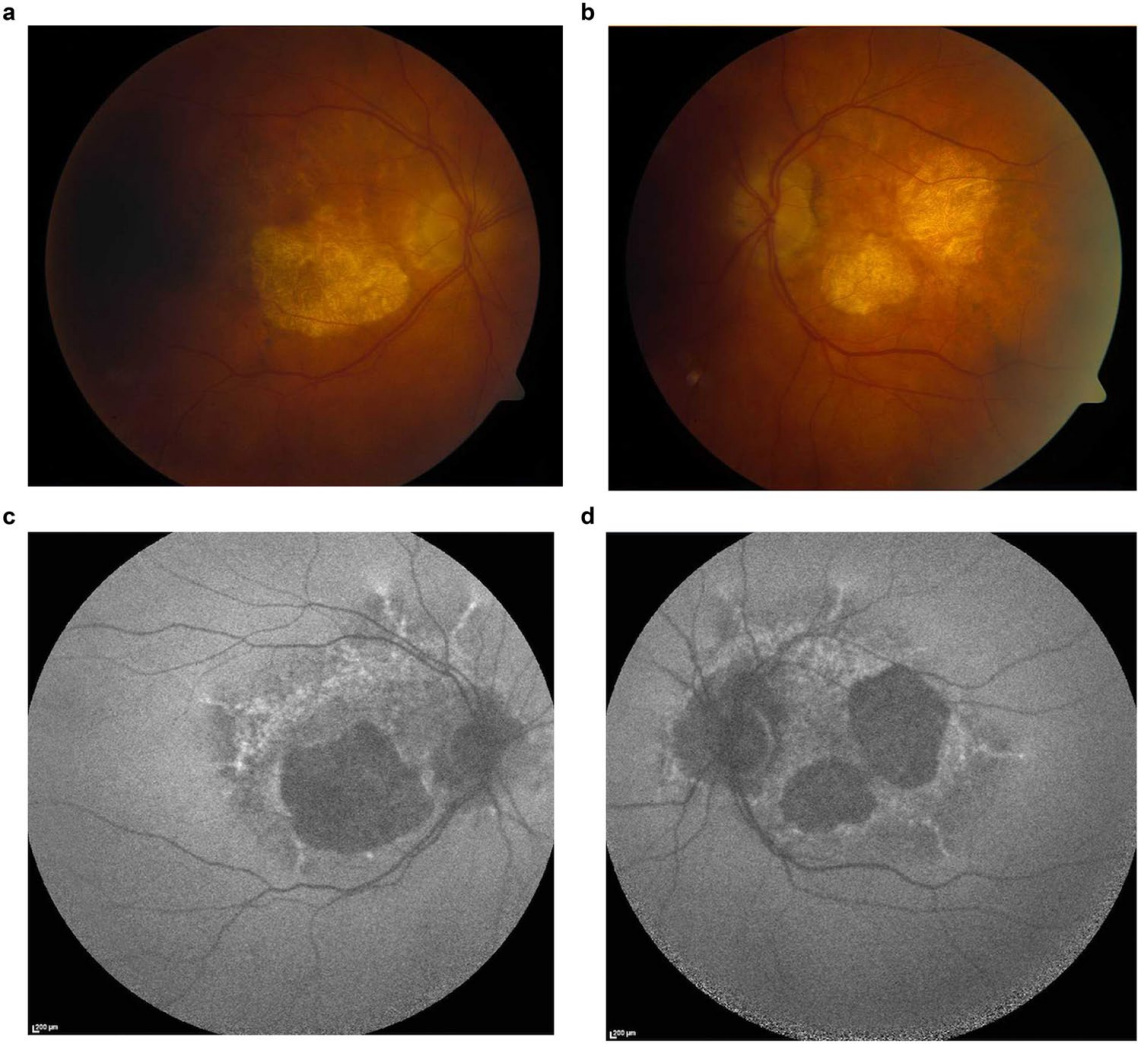
Fundal photographs and autofluorescent images of Patient 1. (**a**,**b**) Colour fundal photographs of Patient 1 showing bilateral parafoveal areas of retinal pigment epithelial atrophy. (**c**,**d**) Corresponding fundus autofluorescent images showing central and paracentral areas of RPE atrophy as black and more extensive areas of stippled hyper-autofluorescence representing areas of lipofuscin build up and altered RPE-photoreceptor exchange across the entire macular area.

hESCs (H9) were obtained from WiCell (Madison, United States). Adult human dermal fibroblasts (Lonza; Basel, Switzerland) used for the generation of the Control hiPSC line (A. Buskin *et al*., manuscript in preparation) were obtained from a healthy 51-year-old male with normal vision and no ocular abnormalities. Dermal fibroblasts were established from two patients known to harbour the m.3243A > G mutation. Patient 1 was a 73-year-old female patient with a 10-year history of bilateral progressive pigmentary macular degeneration, with sparing of the left foveal region and reduced visual acuities of 6/60 in her right eye and 6/9 in her left eye (Fig. 8). In addition to pigmentary retinopathy, Patient 1 had previously been diagnosed with insulin-treated type 2 diabetes mellitus and bilateral sensorineural hearing loss. She did not have any significant neurological deficits. Patient 2 was a 62-year-old male patient with extensive bilateral macular geographic atrophy and his visual acuities were reduced to 6/36 in both eyes. He also suffered from insulin-treated type 2 diabetes mellitus and bilateral sensorineural hearing loss, but there were no documented neurological deficits. This study experimental protocol was approved by the Newcastle University review board and was conducted in accordance with the ICH Good Clinical Practice having obtained written informed consent from the patients. Patient samples were collected according to the “Genotype and phenotype in inherited neurodegenerative diseases” study protocol (13/YH/0310) which was approved by the NRES Committee Yorkshire & The Humber - Bradford Leeds.

### Induced pluripotent stem cell generation and culture

hiPSCs were derived on a layer of mouse embryonic fibroblasts and were subsequently adapted to feeder-free culture conditions on matrigel prior to their characterisation. The methods for all of the above are described in a previous publication^60^. hPSCs were maintained in mTeSR™1 medium (STEMCELL technologies; Vancouver, Canada). The medium was changed daily. The cells were passaged 1:6 with Versene® (EDTA) 0.02% solution (Lonza). For all cell culture experiments with MELAS cells 0.05 mg/ml uridine (Sigma; St. Louis, United States) was added to the medium.

### Retinal pigment epithelium differentiation and expansion

For the RPE differentiation experiments, hPSCs on matrigel in 6-well plates were grown to high density, ensuring that the cells formed a confluent monolayer. Then the medium was changed to Advanced RPMI 1640 Medium (AdRPMI) (Thermo Fisher Scientific; Waltham, United States) with B-27® Supplement (50X) (B27) (Thermo Fisher Scientific), 10% Knock-Out Serum Replacement (Thermo Fisher Scientific), 1% 100 X GlutaMAX (Thermo Fisher Scientific), and 1% Penicillin-Streptomycin Solution (Thermo Fisher Scientific). RPE differentiation medium was replaced every day for the first 3 weeks and twice a week thereafter. Differentiation continued for 3–4 months. When pigmented areas reached 3–5 mm in size, they were mechanically dissected under a stereomicroscope using a 10 µl pipette tip, dissociated with TrypLE™ Select Enzyme (10X) (Thermo Fisher Scientific), filtered through a 40 µm cell strainer (Thermo Fisher Scientific) and re-plated for enrichment and maturation at a density of 4.5 × 10^5^ cells per cm^2^ on plates or 0.33 cm^2^ PET hanging cell culture inserts (Merck Millipore; Billerica, United States) pre-coated with growth factor reduced matrigel (Corning; Corning, United States).

### *In vitro* differentiation into three germ layers

In order to assess the ability of hiPSCs to spontaneously differentiate into cell types representative of the three embryonic germ layers, spontaneous differentiation of cells as a monolayer was performed. Cells were let to grow to a normal passaging density and the medium was changed to DMEM/F-12 (Thermo Fisher Scientific), 20% Fetal Bovine Serum (FBS) (Thermo Fisher Scientific), 1% Penicillin–Streptomycin Solution (Thermo Fisher Scientific) and 1% MEM Non-essential Amino Acids Solution (Thermo Fisher Scientific). Cells were cultured for 2 weeks, replacing the medium daily. Cells were then collected with a cell scraper, washed and stored as pellets at −80 °C until further use.

### DNA extraction

DNA was isolated using the QIAamp DNA Mini Kit (Qiagen; Hilden, Germany), following the instructions of the manufacturer. The purity of the DNA was checked with NanoDrop 2000 spectrophotometer expecting the A260/A280 ratio to be ~1.8. Isolated DNA was stored at −20 °C.

### RNA extraction and cDNA synthesis

RNA was isolated using ReliaPrep™ RNA Cell Miniprep System (Promega; Madison, United States) following the instructions of the manufacturer. The purity of RNA was confirmed using NanoDrop 2000 spectrophotometer expecting the A260/A280 ratio to be ~2.0. cDNA was synthesised subsequently to RNA extraction from 1 mg of total RNA in 40 µl volume using GoScript™ Reverse Transcription System (Promega) according to the manufacturer’s instructions.

### Pyrosequencing experiments

Primers for PCR and pyrosequencing were designed using PyroMark® Assay Design SW 2.0 (Qiagen). PCR was set up with 50 ng DNA template. PCR was a 25 μl MyTaq™ HS DNA Polymerase reaction (Bioline; London, United Kingdom) with 5x MyTaq Reaction Buffer (3 mM MgCl2, 1 mM dNTP), 0.4 µM forward and reverse primers, 5 U/µl MyTaq HS DNA Polymerase and dH_2_O. The following PCR program was used: denaturation 95 °C 15 seconds, annealing 65 °C 30 seconds, elongation 72 °C 10 seconds for 30 cycles. The primer sequences have been provided in Supplementary Table S1. The products were analysed by standard 2.5% agarose gel electrophoresis. Assay set up was created using PyroMark® Q24 Software (Qiagen). Pyrosequencing was completed on PyroMark Q24 (Qiagen) according to the manufacturer instructions. The results were analysed using PyroMark® Q24 Software.

### Quantification of mtDNA copy number

mtDNA was quantified using CFX96 Touch™ Real-Time PCR Detection System (Bio-Rad; Hercules, United States). Reaction volume was 20 µl containing iTaq™ Universal Probes Supermix (Bio-Rad), 0.3 µM forward and reverse primers and sample DNA. Standard curves were used in order to ensure reaction efficiency and accurate quantification. Standard PCR reactions were run for each primer set to generate templates covering the amplification region of the qPCR. PCR reaction conditions were as follows: initial denaturation at 95 °C for 15 seconds, denaturation at 95 °C for 15 seconds, annealing and extension at 61 °C for 15 seconds, elongation at 72 °C for 10 seconds for 30 cycles. The products were run on 1% agarose gel. The bands were dissected out under the UV transilluminator (Gel Doc-II Imaging system) and the product extracted and purified using QIAquick Gel Extraction Kit (Qiagen) according to the manufacturer’s instructions. The amount of total DNA was quantified with NanoDrop 2000 spectrophotometer and mtDNA copy number was calculated using the following equation: mtDNA copy number = [C ÷ (L × 2 × 330)] × A, where C – total DNA concentration (10^−9^ nL), L – amplicon length (bp), A – Avogadro’s number. Once copy number was calculated, the templates were serially diluted with dH_2_O to generate standard curves with 1 × 10^9^ to 1 × 10^1^ copies. Quantitative PCR was performed in triplicate reactions using iTaq™ Universal Probes Supermix system (Bio-Rad). The data were analysed using the Bio-Rad CFX Manager software (Bio-Rad). mtDNA was quantified using the 2^−∆∆Ct^ method, where copy number equals 2 × (2^−∆∆Ct^). *β2* *M* was used as a nuclear reference gene. The sequences for the probes and primers that were used have been provided in Supplementary Tables S2, S3, S4.

### Analysis of gene expression

Quantitative PCR was performed in triplicate reactions using GoTaq® qPCR Master Mix reagent system (Promega). The reaction was run on the Applied Biosystems® QuantStudio™ 7 Flex Real-Time PCR System (Thermo Fisher Scientific). The data was analysed using the QuantStudio™ software (Thermo Fisher Scientific) and relative gene expression was determined using the 2^−∆∆Ct^ method using *GAPDH* as a housekeeping gene. List of primers can be found in Supplementary Table S5.

### Phagocytosis of photoreceptor outer segments

Bovine POS were obtained from InVision BioResources (Seattle, United States). Prior to performing the assay, POS were FITC labelled using the following procedure. The POS were centrifuged at 4500 × g for 4 minutes. They were then re-suspended in AdRPMI, 10% FBS with 0.4 mg/ml FITC (Sigma) and incubated for 1 hour at room temperature protected from light. This was followed by another centrifugation for 4 minutes at 4500 × g. POS were washed three times with PBS and then re-suspended in 2.5% sucrose (Sigma) in PBS and stored in −80 °C until further use. For phagocytosis experiments, normal RPE cell medium was changed to the POS medium (AdRPMI, B27, 10% FBS). Once thawed, POS were re-suspended in POS medium. 1 × 10^6^ FITC-labelled POS were added per cell culture insert for 4 hours at 37 °C. In parallel to that, a negative control experiment was set up when cells were kept for the same time duration but at 4 °C. The incubations were followed by two cell washes with PBS. Cells were then dissociated with TrypLE™ Select Enzyme (10X) (Thermo Fisher Scientific) and washed. They were then re-suspended in 2% FBS solution in PBS with the addition of DRAQ5 (1:400, BioStatus; Shepshed, United Kingdom) for 5 minutes. Extracellular fluorescence was quenched with 0.2% Trypan Blue Stain (Thermo Fisher Scientific) for 10 minutes. Cells were then washed at least three times with PBS and re-suspended in 2% FBS solution in PBS. Cells were analysed on BD™ LSR II flow cytometer (BD Biosciences; Franklin Lakes, United States) collecting at least 10 000 events per sample. The data was analysed on BD FACSDiva software (BD Biosciences).

### Flow cytometry

Pluripotency marker expression was quantified with flow cytometric analysis. Single cell suspension was made with Versene® (EDTA) 0.02% solution and cells were fixed with 2% paraformaldehyde (PFA) for 10 minutes at 37 °C. Cells were then washed with PBS and re-suspended in ice cold methanol. They were then stored at −20 °C for at least 30 minutes and for up to 1 month. For the quantification of the expression of pluripotency markers, cells were washed with PBS, 1 × 10^6^ cells/ml were re-suspended in 100 μl of 2% FBS in PBS with the appropriate concentrations of antibodies, Anti-TRA-1-60, FITC conjugate (Merck Millipore; dilution 1:5) and Nanog XP® Rabbit mAb Alexa Fluor® 647 Conjugate (New England Biolabs; dilution 1:50). Cells were then incubated for 40 minutes on a shaker at room temperature. Cells were washed using BD FACS™ Lyse Wash Assistant (BD Biosciences) and analysed on BD FACSCanto™ II system (BD Biosciences). Data was analysed on BD FACSDiva software (BD Biosciences). A minimum of 10 000 events were recorded for each sample. Gating strategy involved using an appropriate isotype control.

### Immunofluorescence

Cells were washed with PBS and then fixed with 4% paraformaldehyde for 15 minutes at room temperature. Cells were then washed three times with PBS and permeabilised with 0.25% Triton-X-100 (Sigma) for 40 minutes at room temperature. Cells were then washed with PBS following by a blocking step with 10% normal goat serum (Invitrogen; Carlsbad, United States) and 1% BSA (Sigma) in PBS for 45 minutes at room temperature. Cells were then stained with primary antibodies [Anti-ZO1, rabbit (Invitrogen; dilution 1:200), Anti-Bestrophin, mouse (Abcam; Cambridge, United Kingdom; dilution 1:500), Anti-Sodium Potassium ATPase (Alexa Fluor® 488), rabbit (Abcam; dilution 1:50), Anti-CRALB, mouse (Source Bioscience; Nottingham, United Kingdom; dilution 1:100)] at 4 °C overnight, followed by incubation with secondary antibodies [Cy™3 AffiniPure Goat Anti-Rabbit IgG (Jackson ImmunoResearch, West Grove, United States; dilution 1:800), Anti-mouse IgG–FITC antibody (Sigma; dilution 1:800)] for 1 hour at room temperature. All the antibodies were diluted in blocking solution. Nuclei were counterstained with DAPI (CyStain® DNA, Sysmex Partec; Görlitz, Germany). Images were taken with Zeiss Axiovert 200 M – Inverted Widefield microscope with Zeiss AxioCam HRm camera and analysed with AxioVision SE64 Rel. 4.9.1 software. All Carl Zeiss Microscopy GmbH.

### Analysis of RPE pigmentation

Method of quantification of the pigmentation yield by RPE cells was modified from a previously published protocol^61^. Area covered by the emerging pigmented RPE foci was measured by taking the images of the 6-well plates with differentiating RPE cells using the Epson Perfection V500 Photo scanner (Seiko Epson Corporation; Nagano, Japan). Images were acquired at a resolution of at least 300 dots per inch at multiple points during differentiation. The images were analysed with ImageJ (NIH, Bethesda, MD). Images were first converted to greyscale and then using threshold tool only isolated pigmented foci were made visible. Total pigmented area was quantified by measuring grey areas using the software obtaining the total area covered by pigment on a 6-well plate.

### Transmission electron microscopy

Cells were fixed with 2% glutaraldehyde and kept at 4 °C. TEM including all the cell processing was performed at Newcastle University Electron Microscopy Research Services.

### Trans-epithelial resistance

TER was measured in order to assess barrier properties and integrity of the hPSC-RPE cells cultured on hanging cell culture inserts. Measurements were carried out with Millicell ERS-2 Voltohmmeter (Merck Millipore). TER of the blank insert with PBS was measured first followed by the resistance measurements of the cells of interest. An average of two recordings for each sample and a blank was taken. In order to calculate the TER values, the recordings for the blank were subtracted from the recordings of the samples. The result was multiplied by the surface area of the insert and data presented as Ωcm^2^.

### Oxygen consumption

Oxygen consumption rate in control and patient fibroblasts and hiPSCs was measured using Seahorse Bioscience XFe96 Extracellular Flux Analyser (Seahorse Bioscience; North Billerica, United States). The experiments were performed in a Seahorse 96-well XF Cell Culture Microplate. hiPSCs were seeded in the presence of 10 µM Y-27632 (Chemdea; Ridgewood, United States) on the microplates pre-coated with matrigel at a density of 4 × 10^4^ cells per well in 200 µl of growth medium 48 hours prior to the experiment. Medium was changed 5 hours after seeding the cells and then 24 hours later. Fibroblasts were seeded at a density of 2.5 × 10^4^ cells per well in 200 µl of growth medium 24 hours prior to the experiment. On the day of the experiment, medium was changed to 175 µl bicarbonate-free DMEM with the appropriate concentrations of glucose (Sigma), L-glutamine (Thermo Fisher Scientific) and sodium pyruvate (Sigma) to maintain the same concentration as in the usual growth medium, pH was adjusted to 7.0–7.4. Cells were incubated at 37 °C for 1 hour prior to starting the assay. First the baseline oxygen consumption rate was measured. Proton leak was measured by the addition of 1.5 µM and 1 µM of oligomycin to hiPSCs and fibroblasts respectively. Maximal capacity respiration was determined by the sequential injection of carbonyl cyanide-ptrifluoromethoxyphenylhydrazone (first injection 0.3 µM and 0.5 µM for hiPSCs and fibroblasts, second injection 0.5 µM and 1 µM for hiPSCs and fibroblasts respectively). Non-mitochondrial respiration (NMR) was measured by the addition of 1 µM and 2 µM of rotenone to the hiPSCs and 1 µM to fibroblasts respectively. Data was corrected by the NMR and normalised to the total protein concentration measured by Bradford method. Data is presented as pmol of oxygen/minute/mg of protein.

### ATP assay

ATP measurements were performed with the CellTiter-Glo® Luminescence Assay (Promega). Cells were seeded on 96-well white microplates (Thermo Scientific) pre-coated with matrigel at a density of 3 × 10^4^ cells per well in 200 μl of cell culture medium. Cells were expanded for 2 weeks. On the day of the assay, cells were incubated for 1 hour 30 minutes at 37 °C in 200 μl of the ATP buffer with either 5 mM glucose (Sigma) or 5 mM 2-Deoxy-D-glucose (Sigma) or 5 mM glucose with 2.5 μg/ml oligomycin (Sigma) or 5 mM 2-Deoxy-D-glucose and 2.5 μg/ml oligomycin. Following incubation, 90 μl of the buffer was removed and 110 μl of the CellTiter-Glo® Reagent Buffer was added. The plate was shaken in the dark for 10 minutes to induce cell lysis and then left in the dark for further 15 minutes. Luminescence was measured with 1000 ms integration time on a Luminoskan Ascent (Thermo Scientific). The signal was normalised to mg of protein as determined by Bradford method.

### Statistical analysis

All data is expressed as mean ± standard error of the mean unless otherwise stated. One-way analysis of variance (ANOVA) was used to assess variation across multiple samples. The test was followed by Tukey’s multiple comparisons test. Representation of significance of p value was as follows: *p ≤ 0.05, **p ≤ 0.01, ***p ≤ 0.001, ****P ≤ 0.0001. All statistical analyses were done using GraphPad Prism Software Inc. (San Diego, USA).

### Data availability

All data generated or analysed during this study are included in this published article (and its Supplementary Information files).

## Electronic supplementary material

Supplementary Information
