# Supplementary material for: Human iPSC disease modelling reveals functional and structural defects in retinal pigment epithelial cells harbouring the m.3243A > G mitochondrial DNA mutation
Source: Sci Rep. 2017 Sep 26;7:12320. doi: 10.1038/s41598-017-12396-2 (PMC5615077; doi:10.1038/s41598-017-12396-2)
Supplement: Supplementary file 1 — Supplementary Information [file 41598_2017_12396_MOESM1_ESM.pdf]

Supplementary information

Human iPSC disease modelling reveals functional and structural defects in retinal pigment epithelial cells harbouring the m.3243A>G mitochondrial DNA mutation

Valeria Chichagova, Dean Hallam, Joseph Collin, Adriana Buskin, Gabriele Saretzki, Lyle Armstrong, Patrick Yu-Wai-Man, Majlinda Lako and David H Steel

Figure S1. TER measurements. No significant difference was found between patient and control samples. Data presented as mean ± SEM (n=3).

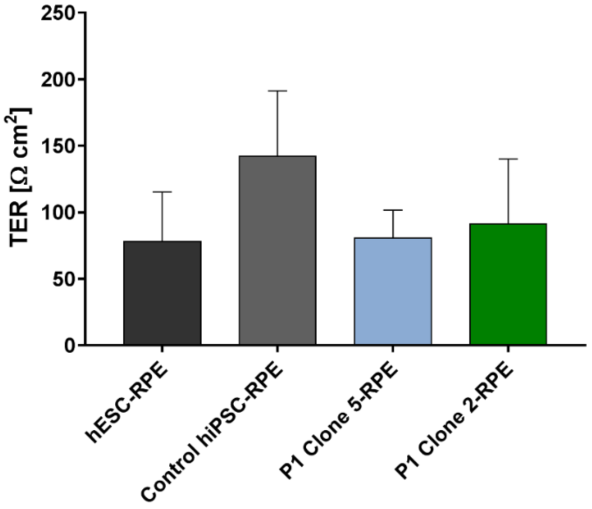

Figure S2. ATP production by OXPHOS and glycolysis. No observable difference between the cells. Data presented as mean ± SD (n=2). RLU – relative light units.

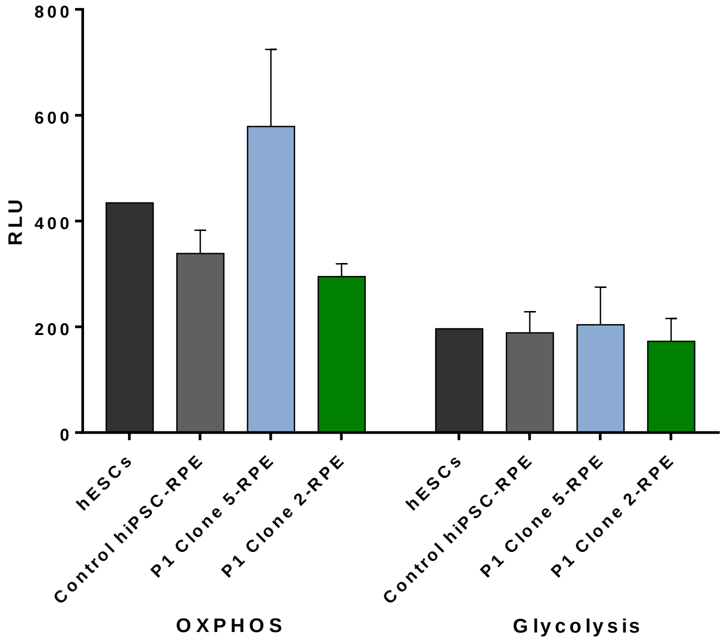

Figure S3. Full length gel images from Figure 2. All gel images were taken with similar exposure levels ensuring none were overexposed. The original gel images had additional samples irrelevant for this project and therefore were excluded from the images shown in Figure 2.

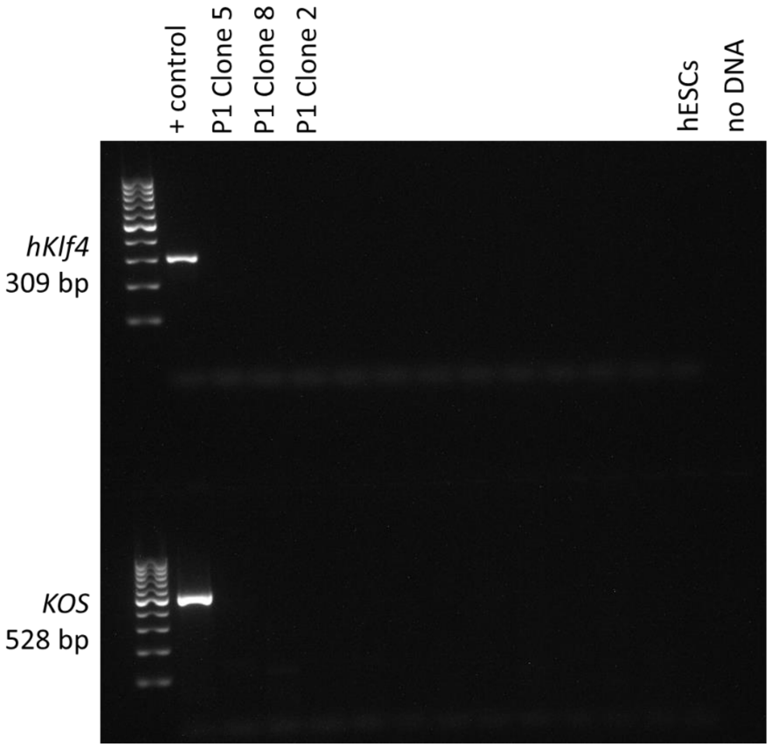

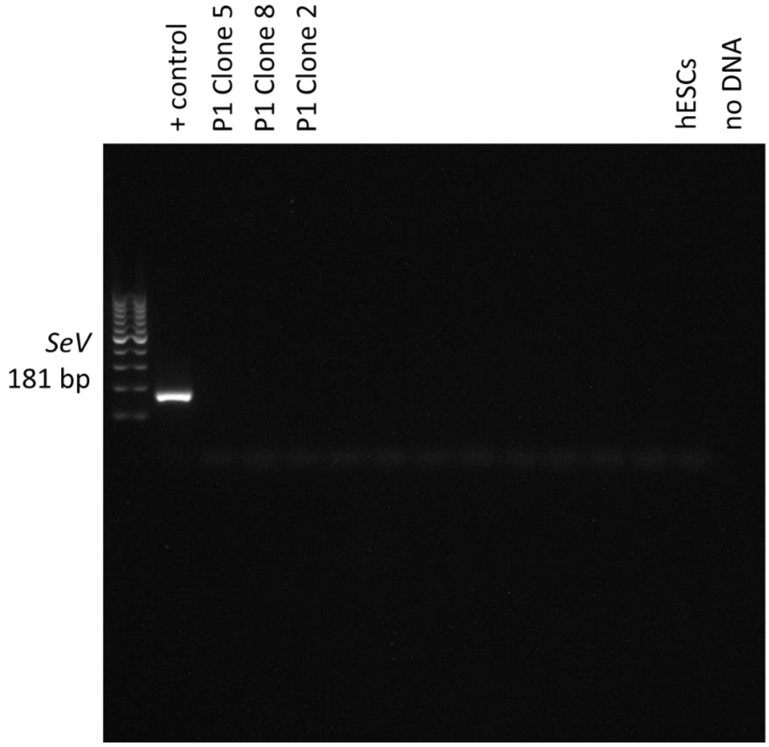

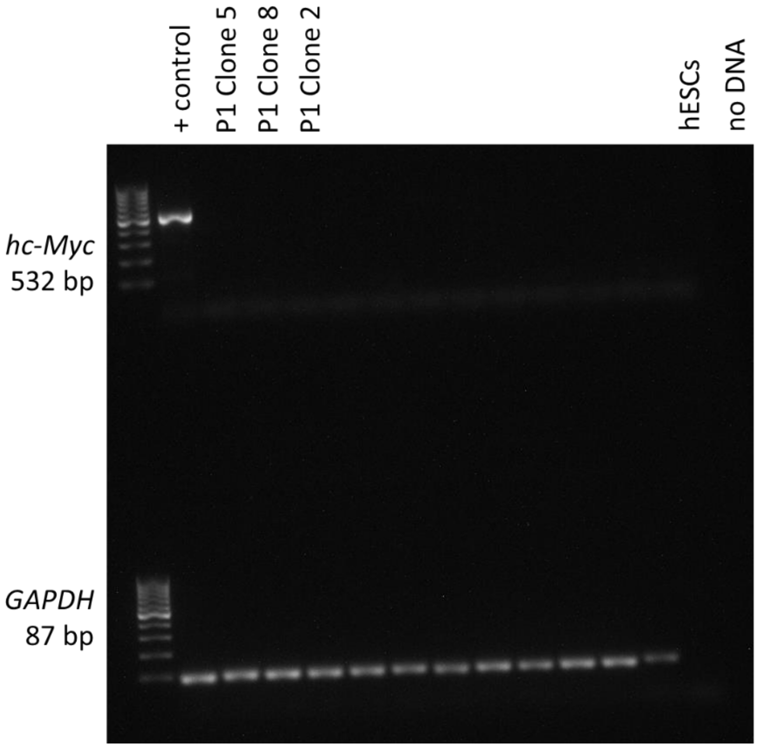

Table S1. Primer sequences for pyrosequencing experiments. BIO – biotinylated.

Target	Primer sequence	Position of primer
m.3243A>G	Forward: *BIO-TAAGGCCTACTTCACAAAGCG	3142-3353
	Reverse: GCGATTAGAATGGGTACAATGAG	3258-3244
	Sequencing: ATGCGATTACCGGGC	

Table S2. Primer sequences for PCR reactions for generation of standards.

Target	Primer sequence	Product size (bp)	Annealing temperature (°C)
β2M	Forward: CGCAATCTCCAGTGACAGAA Reverse: GCAGAATAGGCTGCTGTTCC	1092	61
MTND1	Forward: CAGCCGCTATTAAAGGTTCG Reverse: AGAGTGCGTCATATGTTGTTC	1040	61

Table S3. qPCR primer sequences for mtDNA copy number.

Target	Primer sequence	Product size (bp)
β2M	Forward: CACTGAAAAAGATGAGTATGCC<br>Reverse: AACATTCCCTGACAATCCC	231
MTND1	Forward: ACGCCATAAAACTCTTCACCAAAG<br>Reverse: GGGTTCATAGTAGAAGAGCGATGG	111

Table S4. Sequences for iTaq probes.

Target	Sequence	Fluorophore	Quencher
β2M	CCGTGTGAACCATGTGACTTTGTC	FAM	BHQ_1
MTND1	ACCCGCCACATCTACCATCACCCTC	HEX	BHQ_1

Table S5. List of primers used for gene expression analyses.

Gene	Forward primer (5'→3')	Reverse primer (5'→3')
CDX2	CTCGGCAGCCAAGTGAAAAC	CTCCTTTGCTCTGCGGTTCT
CRALBP	ACCTTTGATGAGATCTTGCAG	GAAGCCATTGATTTGAGTTTCC
FOXA2	GCATTCCCAATCTTGACACGGTGA	GCCCTTGCAGCCAGAATACACATT
GAPDH	TGCACCACCAACTGCTTAGC	GGCATGGACTGTGGTCATGAG
MERTK	AGCCTGAGAGCATGAATGTCACCA	TGTTGATCTGCACTCCCTTGGACA
MITF	GCCTCCAAGCCTCCGATAAG	CATCTGCTCACGCATGAGTTG
MIXL	GAGACTTGGCACGCCTGT	GGTACCCCGACATCCACTT
NANOG	AGAAGGCCTCAGCACCTAC	GGCCTGATTGTTCCAGGATT
OA1	AGCATGCTGTCTGAAGATGAGA	GTGTCTGTGTCCTAGCAGGTT
OTX2	CAAAGTGAGACCTGCCAAAAAGA	TGGACAAGGGATCTGACAGTG
PAX6	GCCTATGCAACCCCCAGT	TCACTTCCGGGAACTTGAAC
RPE65	GCCCAGGAGCAGGACAAAAG	GCGCATCTGCAAGTTAAAACCA
SILV	GGGCCCCCTGCTGGATGGTA	CCCGCCTTGGCAGGACACAG
SOX1	GGAATGGGAGGACAGGATTT	ACTTTTATTTCTCGGCCCGT
T	CAGTGGCAGTCTCAGGTTAAGAAGGA	CAGTGGCAGTCTCAGGTTAAGAAGGA
TYR	TAGCGGATGCCTCTCAAAGC	CAATGGGTGCATTGGCTTCT
